# Neurological activation during verbal fluency task and resting-state functional connectivity abnormalities in obsessive-compulsive disorder: a functional near-infrared spectroscopy study

**DOI:** 10.3389/fpsyt.2024.1416810

**Published:** 2024-08-30

**Authors:** Yongjun Qiao, Xiaohui Song, Jin Yan, Wenxiu Pan, Chinhsuan Chia, Dan Zhao, Chuanxin M. Niu, Qing Xie, Haiyan Jin

**Affiliations:** ^1^ Department of Rehabilitation Medicine, Ruijin Hospital, Shanghai Jiao Tong University School of Medicine, Shanghai, China; ^2^ School of Medicine, Shanghai Jiao Tong University, Shanghai, China; ^3^ Department of Psychiatry, Ruijin Hospital, Shanghai Jiao Tong University School of Medicine, Shanghai, China

**Keywords:** obsessive-compulsive disorder, functional near-infrared spectroscopy, verbal fluency task, functional connectivity, brain activation

## Abstract

**Objective:**

This study aims to investigate the activation of frontotemporal functional brain areas in patients with Obsessive-Compulsive Disorder (OCD) during a Verbal Fluency Task (VFT), and to compare their brain functional connectivity in a resting state with that of healthy controls. The goal is to deepen our understanding of the neuropathological mechanisms underlying OCD.

**Methods:**

32 patients with OCD and 32 controls matched for age, gender, handedness, and years of education participated in this study, they were divided into OCD group and healthy comtrol group. We conducted VFT task tests and 10-minute resting state tests on both groups by using functional Near-Infrared Spectroscopy (fNIRS). The VFT was utilized to assess the activation (beta values) and the integral and centroid values of the frontal and bilateral temporal lobes, including brain areas BA9 and 46 (dorsolateral prefrontal cortex), BA10 (frontal pole), BA45 (inferior frontal gyrus), BA21 (middle temporal gyrus), and BA22 (superior temporal gyrus). We evaluated the functional connectivity levels of these areas during the resting state. Differences in these measures between OCD patients and healthy controls were analyzed using two-sample independent t-tests and non-parametric Mann-Whitney U tests.

**Results:**

During VFT, OCD had smaller integral values(*z*=5.371, *p*<0.001; *t*=4.720, *p*<0.001), and larger centroid values(*t*=-2.281, *p*=0.026; *z*=-2.182, *p*=0.029) compared to healthy controls, along with a reduced number of activated channels detected by fNIRS. Additionally, activation values (β) in various functional brain areas, including BA9, BA46, BA10, BA45, BA21, and BA22, were significantly lower in the OCD group (all *p*< 0.01). In the resting state, notable disparities in functional connectivity were observed between the inferior frontal gyrus (IFG) and dorsolateral prefrontal cortex (DLPFC) in the OCD group, in comparison to the control group. Specifically, there was a significant increase in connectivity between the left IFG and right DLPFC, suggesting the presence of altered connectivity patterns in these areas.

**Conclusions:**

The study highlights significant disparities in neural activation and functional connectivity between OCD patients and healthy controls during VFT. Specifically, reduced activation was noted in the frontal and bilateral temporal lobes of OCD patients, alongside alterations in resting-state functional connectivity between the IFG and DLPFC. These findings contribute to our understanding of the neurobiological underpinnings of OCD and may guide future therapeutic strategies.

## 1 Introduction

Obsessive-compulsive disorder (OCD) is a prevalent mental health condition encountered in clinical settings, characterized by persistent, unwanted thoughts and/or repetitive behaviors that lead to significant distress and are difficult for the individual to control (1). With a lifetime prevalence of approximately 2.3%, OCD often manifests during adolescence (2). Unfortunately, many affected individuals do not receive timely diagnosis and treatment, contributing to the chronic nature of the disorder and imposing considerable socio-economic burdens (3).

Understanding the etiology of OCD is crucial for advancing prevention and treatment strategies. Although the serotonin hypothesis has been a longstanding focus (4), its specific pathophysiological role remains unconfirmed, as evidenced by mixed results from randomized clinical trials involving serotonergic medications (5). More recently, the glutamate hypothesis has garnered support; yet, the precise implications of glutamate dysregulation in OCD are still to be fully elucidated (6). With the advancement of neuroimaging techniques, research on OCD has increasingly focused on the use of functional imaging studies such as functional magnetic resonance imaging (fMRI) and positron emission tomography (PET). Consistently, these studies have identified hyperactivity in the cortico-striato-thalamo-cortical(CSTC) circuit (7–9) and abnormalities in the orbitofrontal cortex, striatum, and caudate nucleus among individuals with OCD (10, 11). However, the limitations of fMRI and PET, particularly in terms of temporal resolution, portability, and patient tolerability (12–14), have underscored the need for alternative imaging modalities.

Functional near-infrared spectroscopy (fNIRS) emerges as a promising tool in this context, offering advantageous spatial and temporal resolution, portability, cost-effectiveness, and robustness against motion and electromagnetic interference (15). These attributes make fNIRS an ideal choice for probing neural activity across both task and resting states (RS) (16), thereby facilitating a comprehensive examination of OCD’s neurobiological mechanisms in spatial and temporal dimensions.

Since the introduction of research exploring the potential of combining fNIRS with VFT as an auxiliary diagnostic method for mental illnesses (17), numerous validation studies have followed. Researchers suggest this paradigm could aid in understanding, detecting, and differentiating mental illnesses, paving the way for neuroimaging biomarkers (18). Concurrently, the Chinese VFT has also demonstrated its feasibility and accuracy in assisting mental illness diagnosis (19). A previous fNIRS study on patients with obsessive-compulsive disorder (OCD) found varying degrees of underactivation in the frontal and temporal lobes, which may be related to abnormalities in language, cognition, and executive functions in OCD patients (20). The Verbal Fluency Task (VFT), leveraging executive functions, is utilized herein due to its effectiveness in eliciting rich data on language capability and executive control. This task, which requires speech retrieval alongside self-monitoring and response inhibition (21), serves as a valuable instrument for assessing cortical cognitive functions (22, 23). Given the cognitive deficits characteristic of OCD and related disorders (24), the VFT represents a viable approach to investigate cortical activity patterns in this population. Preliminary applications of fNIRS in pediatric OCD research indicated a diminished prefrontal cortex hemodynamic response during cognitive tasks (25). Subsequent adult studies confirmed these findings, particularly noting reduced activity in the right dorsolateral prefrontal cortex (DLPFC) during the VFT (26). While both temporal and frontal lobes’ decreased activation levels were associated with symptom severity, the research also unveiled variability in DLPFC functionality among OCD patients presenting with different symptoms. Such inconsistencies underscore the necessity for further inquiry into OCD’s effects across various brain areas (27, 28).

In parallel, fMRI studies have highlighted the diagnostic potential of resting-state functional connectivity (RSFC) patterns in mental disorders, linking them to specific cognitive and psychiatric conditions (29–32). The emergence of fNIRS has prompted comparisons with fMRI-RSFC, revealing substantial congruence and affirming fNIRS’s reliability and validity for RSFC analysis. Despite these advancements (33–37), fNIRS-based research on OCD’s resting state remains scarce, warranting this study’s exploration of RSFC differences in OCD patients.

By harnessing fNIRS to combine VFT and RSFC analyses, this investigation seeks to elucidate the neural mechanisms of OCD, focusing on both brain activation during task performance and network connectivity in resting states. The objective is to deepen our understanding of the neurobiological underpinnings of OCD, contributing to the broader field of psychiatric neuroscience.

## 2 Materials and methods

### 2.1 Participants

This study enrolled a cohort of 39 individuals diagnosed with OCD, recruited from Ruijin Hospital, which is affiliated with the Shanghai Jiaotong University School of Medicine. In parallel, 36 healthy individuals were selected to serve as control subjects. After data quality control, 7 patients of OCD and 4 healthy individuals were excluded because of inclusion, exclusion criteria or incomplete collection. The final sample comprised 32 patients and 32 healthy controls (**Table 1**). Prior to participation, all participants were provided with detailed information about the study and gave their informed consent by signing a consent form, in accordance with the ethical standards set forth in the Declaration of Helsinki. This study protocol was approved by the Institutional Review Board of Shanghai Jiaotong University Ruijin Hospital (NO.322 of 2022).

**Table 1:** Comparison of clinical characteristics between HC group and OCD group.

Indicators	HC(n=32)	OCD(n=32)	*χ2*	*z*	*t*	*p*
Male	14	19	1.564			0.371
Female	18	13				
Age(years)	22.50(21.00,26.50)^a^	22.00(18.00,28.75)		0.653		0.514
Education(years)	15.00(14.25,16.00)	14.50(11.25,16.00)		1.872		0.061
Y-BOCS scores	3.25 ± 1.44 ^b^	17.41 ± 6.11			-12.756	0.001^*^
SSS score	2.00(1.25,3.00)	2.50(2.00,3.00)		-0.478		0.632

^a^Represented by the interquartile range (IQR) as [M (Q1, Q3)]; ^b^Mean ± SD; ^*^p< 0.05.

The inclusion criteria for the OCD group were as follows:

1. Diagnosis of OCD based on the Diagnostic and Statistical Manual of Mental Disorders, Fourth Edition (DSM-IV) criteria, confirmed by a psychiatrist at the level of deputy director or higher;
2. Right-handed, as assessed by the Edinburgh Handedness Inventory (EHI);
3. Aged between 14 and 60 years;
4. Education level of junior middle school or higher;

No abnormalities detected in head MRI scans;6. Yale-Brown Obsessive-Compulsive Scale (Y-BOCS) score of ≥ 8 and Stanford Sleepiness Scale (SSS) score of < 4 at the time of group entry.

The inclusion criteria for the healthy control group were as follows:

1. No history and chief complaint of any psychiatric or neurological disorders;
2. Right-handed, as assessed by the Edinburgh Handedness Inventory (EHI);
3. Aged between 14 and 60 years;
4. Education level of junior middle school or higher;

No abnormalities detected in head MRI scans;6. Yale-Brown Obsessive-Compulsive Scale (Y-BOCS) score of ≥ 8 and Stanford Sleepiness Scale (SSS) score of < 4 at the time of group entry.

The exclusion criteria for the OCD and healthy control group were:

1. Diagnosis of schizophrenia, mood disorders, depression, or any other mental disorders as defined by DSM-IV criteria; presence of severe neurological diseases, organic mental disorders, severe somatic diseases, or history of craniocerebral trauma;
2. Communication difficulties;
3. Left-handedness (as determined by the EHI);
4. Women who were pregnant or lactating;
5. Dependence on drugs, psychoactive substances, or alcohol;
6. Stanford Sleepiness Scale (SSS) score of ≥ 4;
7. Inability to complete the fNIRS examination due to severe cognitive impairment.

### 2.2 Task design

**Figure 1:**
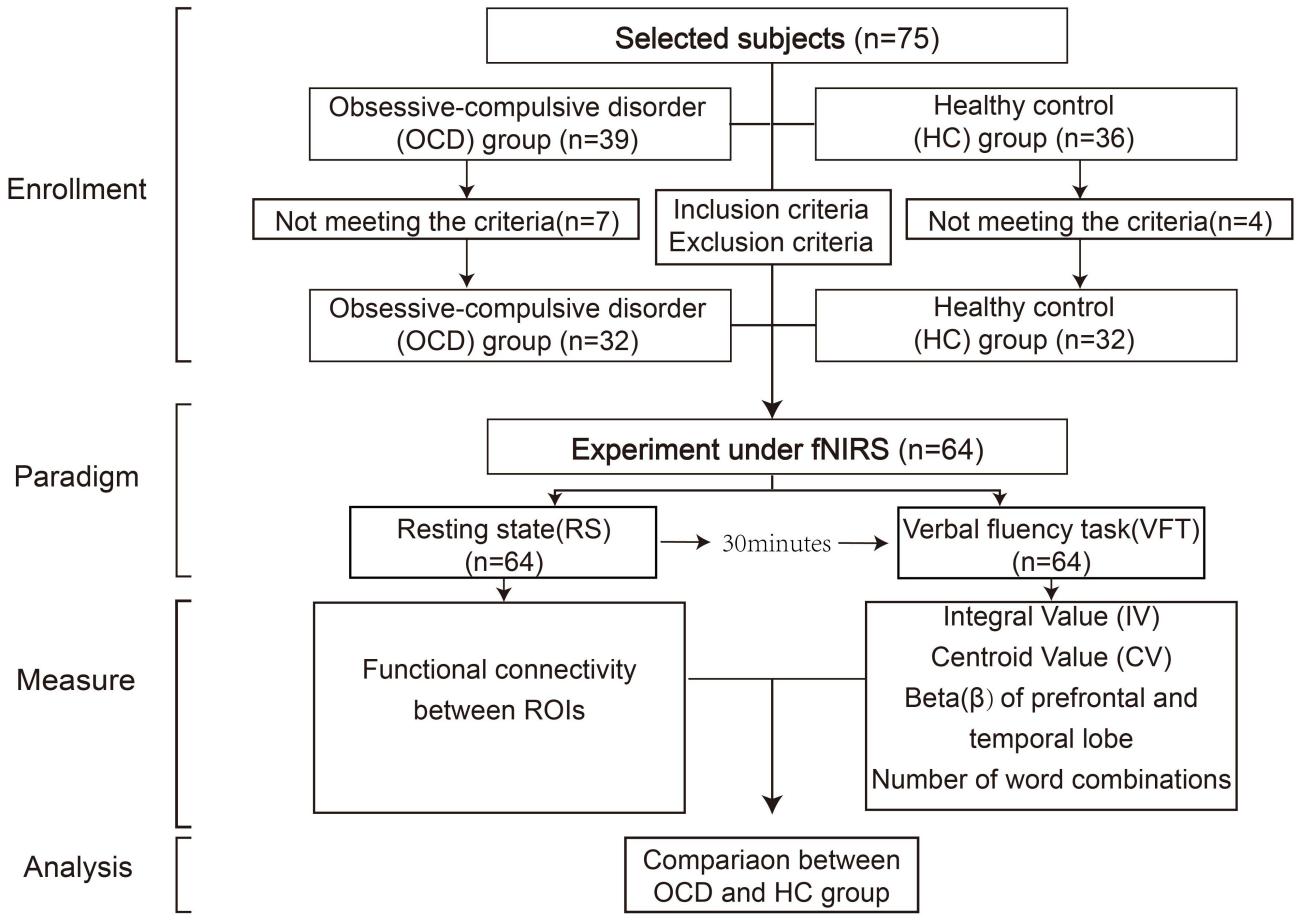
Flow chart of experiment.

All the subjects, including the OCD group and the healthy control group, were tested in the same room, including resting-state test and VFT test. To avoid unnecessary impacts of the VFT on resting-state activities, we chose to let the subjects take the resting-state test first. The participants remained in the same room that was used for the resting-state (RS) task and were given 30 minutes to rest. After the 30-minute break, the VFT test was conducted. The entire experimental flow is depicted in **Figure 1**.

#### 2.2.1 Resting-state (RS) task

**Figure 2:**
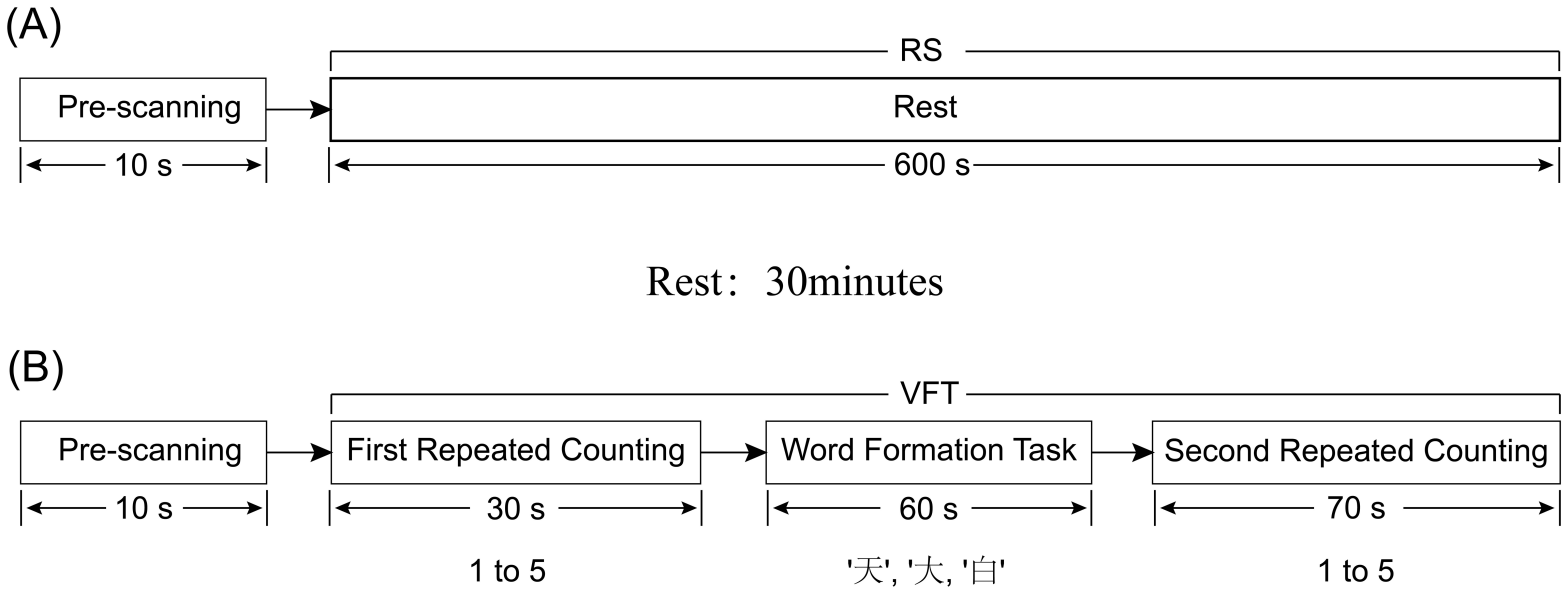
Diagram of RS and VFT tasks, **(A)** represents the RS task process; **(B)** represents the VFT task process.

The resting-state task was administered in a controlled, quiet setting to minimize external distractions. They were allowed to lean on the chair to minimize any unnecessary movements. The RS testing phase was organized into two stages: the first stage, a pre-scanning period lasting for 10 seconds, involved subjects focusing and preparing for the commencement of the test; the second stage, the main test period, required participants to close their eyes and rest for 10 minutes, ensuring they remained awake throughout (**Figure 2A**).

#### 2.2.2 Verbal fluency task (VFT)

After the rest period, the participant was asked to sit comfortably in a chair, with a screen featuring a black cross in the center placed approximately one meter in front of them. They were instructed to keep their eyes open, fixate on the cross to minimize head movements, and to listen carefully to the instructions provided before the start of the task to ensure uniform understanding across participants (**Figure 2B**). During the VFT word formation task, the total number of word phrases generated by each subject for the three Chinese characters will be recorded.

The VFT was structured into four sequential stages, spanning a total of 160 seconds:

- Pre-scanning Period (10 seconds): Participants were asked to focus and prepare for the task’s commencement, creating a baseline period of calm and concentration.
- First Repeated Counting Period (30 seconds): Participants repeated the numerical sequence from 1 to 5 aloud.
- Word Formation Task (60 seconds): During this central phase, participants were presented with three simple Chinese characters (‘天’, ‘大’, ‘白’) via an audio prompt. For each character, allocated 20 seconds, participants were required to continuously form words within 20 seconds until prompted for the next word tasks. The characters were presented in a fixed order, and all participants received the same characters without variation.
- Second Repeated Counting Period (70 seconds): At this final stage, the subjects received the same counting instructions as they did at the beginning. Participants once again repeated the sequence from 1 to 5 aloud. This served to transition participants out of the cognitive engagement of the word formation task.

### 2.3 fNIRS data acquisition

#### 2.3.1 fNIRS instrument

**Figure 3:**
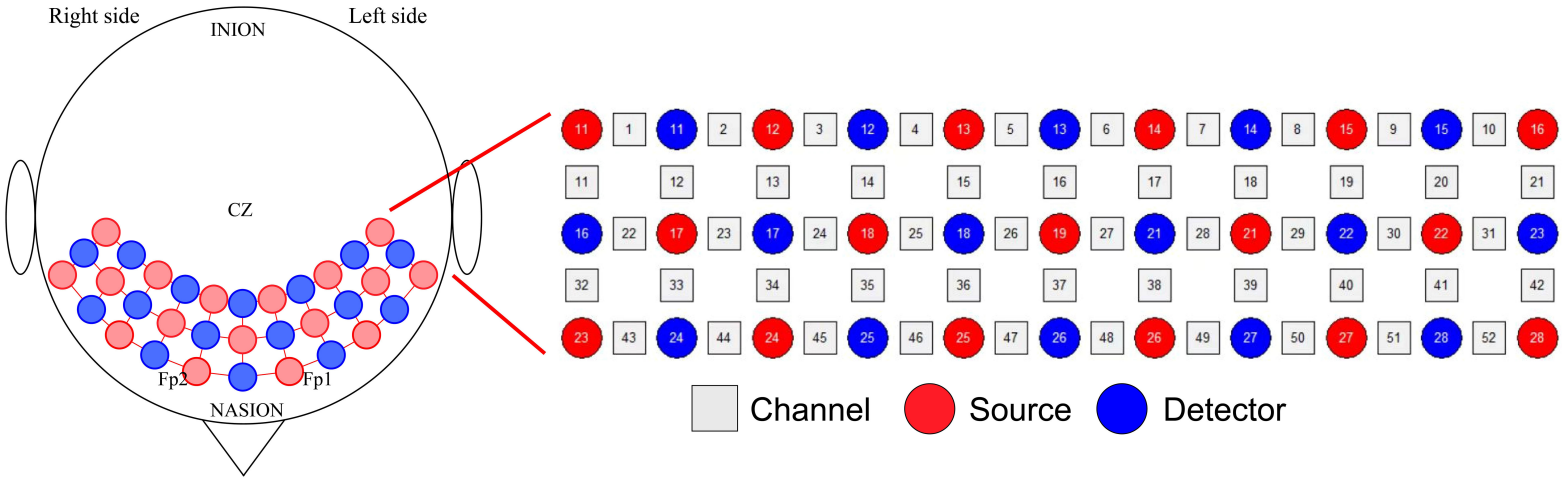
fNIRS 3 × 11 photoelectrodes.

**Figure 4:**
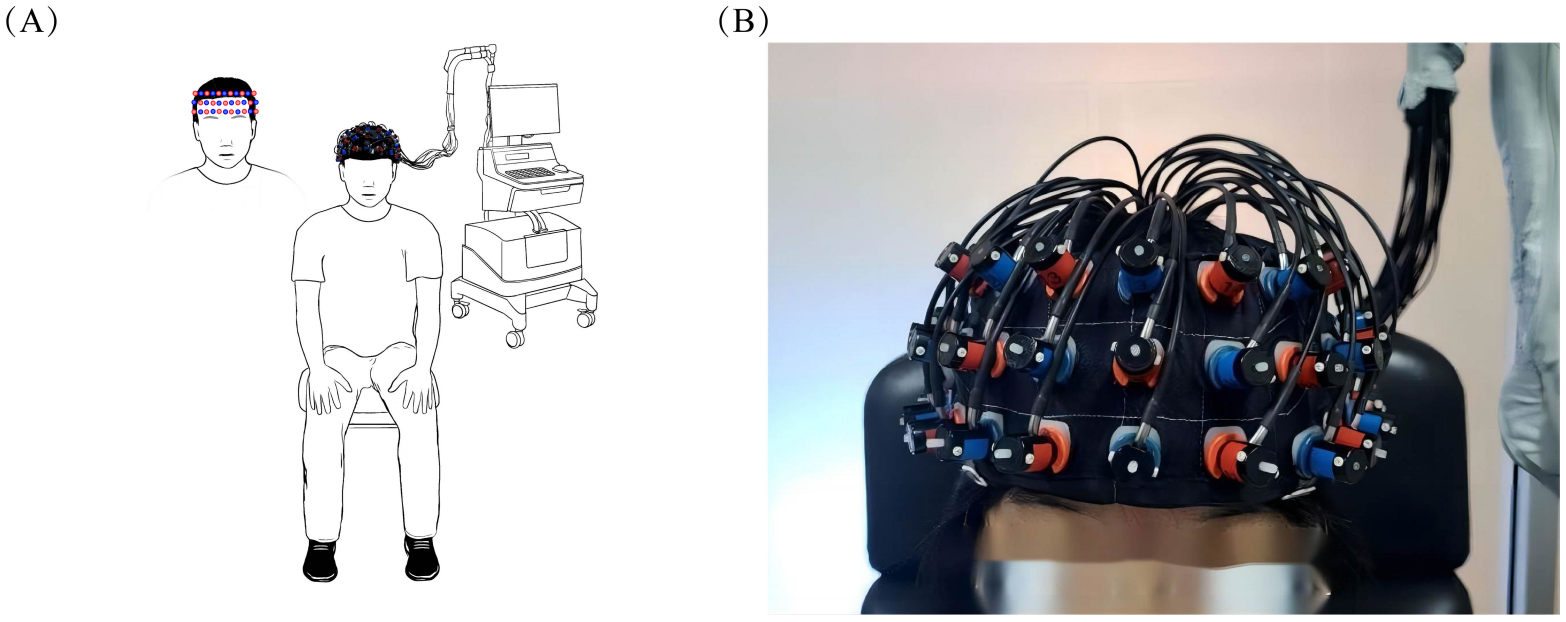
Placement of fNIRS optical cover plate. **(A)** demonstrates the overall appearance of the subject wearing the fNIRS optical cover plate in the room, while **(B)** provides a detailed illustration of the specific placement area of the fNIRS optical cover plate.

Brain activation was assessed using the ETG-4100, a 52-channel multichannel fNIRS instrument by Hitachi Medical Co., Japan, at the Rehabilitation Medicine Department of Ruijin Hospital, affiliated with the Shanghai Jiaotong University School of Medicine. This setup consisted of 17 sources and 16 detectors. Each adjacent pair of a source and detector formed one of the total 52 channels, with a 3cm distance maintained between each source and detector. The instrument’s sampling rate was set at 10Hz. The configuration of the photoelectrodes was arranged in a 3 × 11 matrix (**Figure 3**), positioned in accordance with the international 10-20 system to cover the prefrontal lobe and bilateral temporal lobes. The No. 26 detector was placed just above the center of the eyebrow arch, ensuring the bottom of the fNIRS optical cover plate was parallel to the eyebrows and aligned with the Fp1-Fp2 line of the international 10-20 system (**Figure 4**). Participants were instructed to remain still, minimizing excessive blinking, chewing, and wide mouth openings during testing. Each source emitted two wavelengths of near-infrared light, 695nm and 830nm, which penetrated the skull. The detector captured the residual light particles, and, following the Beer-Lambert Law, the imaging system processed the optical signals into electrical ones to calculate the relative concentrations of oxygenated (oxy-Hb) and deoxygenated hemoglobin (deoxy-Hb).

#### 2.3.2 Data indicators and processing

Following the analysis by the imaging system, we observed relative changes in the cerebral cortical concentrations of oxygenated hemoglobin (oxy-Hb), deoxygenated hemoglobin (deoxy-Hb), and total hemoglobin (t-Hb) during the RS and VFT. Area-specific brain activation leads to increases in both regional cerebral blood flow (rCBF) and the regional cerebral metabolic rate of oxygen. Notably, changes in oxy-Hb are congruent with those in CBF, whereas changes in deoxy-Hb correlate with alterations in the oxygenation and volume of venous blood. It’s important to highlight that minor variations in CBF do not necessarily result in changes in t-Hb. Consequently, oxy-Hb emerges as the most sensitive marker for detecting shifts in rCBF within the context of fNIRS measurements (38). Our analysis primarily concentrated on oxy-Hb (39). We analyzed converted hemoglobin concentration change data using the NIRS-KIT software package in MATLAB R2013b (The MathWorks Inc., Natick, MA, USA) (40). RS and VFT data seamlessly integrated with NIRS-KIT’s supported format directly sourced from Hitachi ETG4000 systems in (.csv) format.

##### 2.3.2.1 RS data processing

We converted RS data into the format supported by NIRS-KIT, then preprocessed the files through initial trimming of the first 20s and last 40s (41), detrending, motion correction with TDDR (42), and bandpass filtering (0.01 to 0.08Hz) (36, 43, 44) using a third-order IIR filter. NIRS-KIT facilitated the definition of networks of interest and subsequent calculation of the FC matrix for these specified networks.

##### 2.3.2.2 VFT data processing

The ETG-4100 instrument automatically conducts integral analysis post-VFT. For this process, we designated the pre-task baseline as the final 10 seconds of the preceding 30-second interval and the post-task baseline as the initial 55 seconds of the subsequent 70-second interval. We get two visual indices that reflectte the temporal evolution of the fNIRS signal: the “Integral Value (IV)” and the “Centroid Value (CV)” by using 5-second moving average approach (17). The IV quantifies the cumulative magnitude of the hemodynamic response throughout the 60-second task activation period. Meanwhile, the CV represents the temporal midpoint of the fNIRS signal change area spanning both the task and post-task periods (19). For analysis purposes, we opted to work with the converted hemoglobin concentration data rather than the raw optical intensity data captured by the near-infrared instrument. This decision was made to preserve the correlation between the IV and CV during post-processing.

Our preprocessing protocol encompassed detrending, motion correction through temporal derivative distribution repair (TDDR) (42), and applying a third-order IIR filter for default band-pass filtering (0.01 to 0.08Hz). We then conducted individual-level statistical analyses to quantify each channel’s activation level during the 60-second task, denoted as β-values.

#### 2.3.3 Functional brain area localization

Given uncontrolled variations in head circumference among subjects, we conducted a brain area localization test for each participant following their session. For post-experimental localization, we employed the Vpen system (Polhemus Patriot, Polhemus Inc., Colchester, VT, USA) to ensure accurate channel-to-functional brain area correspondence (45). The scalp positioning function of the Vpen system enables us to locate the fNIRS cap channels for each patient after the experiment. The Vpen system returns a spatial positioning information file based on the position of the positioning pen on the scalp, which includes “origin” and “others” files. The “origin” file contains the actual coordinates of the reference points; while the “others” file contains the actual coordinates of the channels and optodes. These files can then be imported into the “Spatial Registration” function of NIRS-KIT for NFRI-based registration.

The NFRI toolbox standardized positioning data of optodes and channels to MNI space, allowing for the output of MNI coordinates for each channel and optode within standard brain space (46). NIRS-KIT provides the following three types of anatomical brain region labels: Anatomical Automatic Labeling (AAL) (47); Brodmann areas; LONI Probabilistic Brain Atlas (LPBA40) (48). And the anatomical partition label and corresponding probability for each optode or channel are provided. We selected anatomical brain region labels from the Brodmann areas, and based on the probability of the channels falling within the BA brain region, we chose those with a probability greater than 0.9 and classified them into the corresponding brain areas for further analysis. Channels with a probability less than 0.9 were considered to potentially not fully match the targeted brain region, and thus were excluded from subsequent analysis.

We chose those with a probability greater than 0.9 and classified them into the corresponding brain areas for further analysis. We selected 23 channels in total, which are distributed in six different BA zones. These BA zones are further divided into left and right zones. The selected brain areas and their corresponding channels are as follows: Right BA9 (CH4, CH5, CH15, CH16), Left BA9 (CH6, CH7, CH16, CH17), Right BA10 (CH37, CH47), Left BA10 (CH37, CH38, CH48), Right BA45 (CH24, CH34, CH45), Left BA45 (CH29, CH40, CH50), Right BA21 (CH43), Left BA21 (CH52), Right BA22 (CH32), Left BA22 (CH42), Right BA46 (CH25), and Left BA46 (CH28). Among them, CH16 and CH37 are placed in the exact middle, so they are attributed to both sides of the brain areas for calculation.

### 2.4 Statistical analysis method

Statistical analyses were conducted using SPSS 22.0. For categorical variables, such as gender, chi-square tests assessed inter-group differences. Continuous variables—age, years of education, and the IV, CV, and β values for each region of interest (ROI)—underwent Shapiro-Wilk tests to confirm normality prior to analyzing differences. Normally distributed data were reported as mean ± standard deviation (mean ± SD), and non-normally distributed measurements were represented by the interquartile range (IQR) as [M (Q1, Q3)]. Blood oxygen data from the resting state analysis underwent full-matrix Pearson correlation after z-transformation and were corrected for the false discovery rate (FDR) using NIRS-KIT. The blood oxygen data obtained during VFT, after being processed by the NIRS-KIT, yields β values that can represent activation. Firstly, we conduct internal statistics on the two groups, using either a one-sample T-test or a non-parametric test to calculate the difference between the β-values and 0, indicating whether there is brain activation within the channel. Then independent sample t-tests and Mann-Whitney U tests examined inter-group variations between OCD and HC group. The index for comparing the groups was the degree of functional connectivity across 23 channels and the β values of 12 specifically selected brain areas. These β values were derived as the mean of the β values within each respective brain area’s channels. A p-value < 0.05 denoted statistical significance.

Furthermore, we have comprehensively reported the effect sizes in our analysis. For data adhering to a normal distribution, we utilize an independent-samples t-test, and calculate Cohen’s d as a measure of effect size, where d values of.8,.5, and.2 correspond to large, medium, and small effect sizes, respectively. Conversely, for data that fail to conform to a normal distribution, we apply the Mann-Whitney U test, and calculate the effect size using r = z/ing where r values of.5,.3, and a smaller value indicate large, medium, and small effects, respectively (49).

## 3 Results

### 3.1 General characteristics of subjects

The comparison between the healthy control (HC) and obsessive-compulsive disorder (OCD) groups revealed no significant differences across all observed indicators. Specifically, chi-square tests indicated no significant difference in sex distribution (*χ2 =* 1.564, *p*=0.371), and Mann-Whitney U tests showed no significant disparities in age [22.50(21.00,26.50) vs 22.00 (18.00,28.75), *z*=0.653, *p*=0.514] or years of education [15.00(14.25,16.00) vs 14.50 (11.25,16.00), *z*=1.872, *p*=0.061] between the two groups. A detailed summary of the general characteristics of the study participants (n = 64) is presented in **Table 1**.

### 3.2 RS functional connectivity analysis results

**Figure 5:**
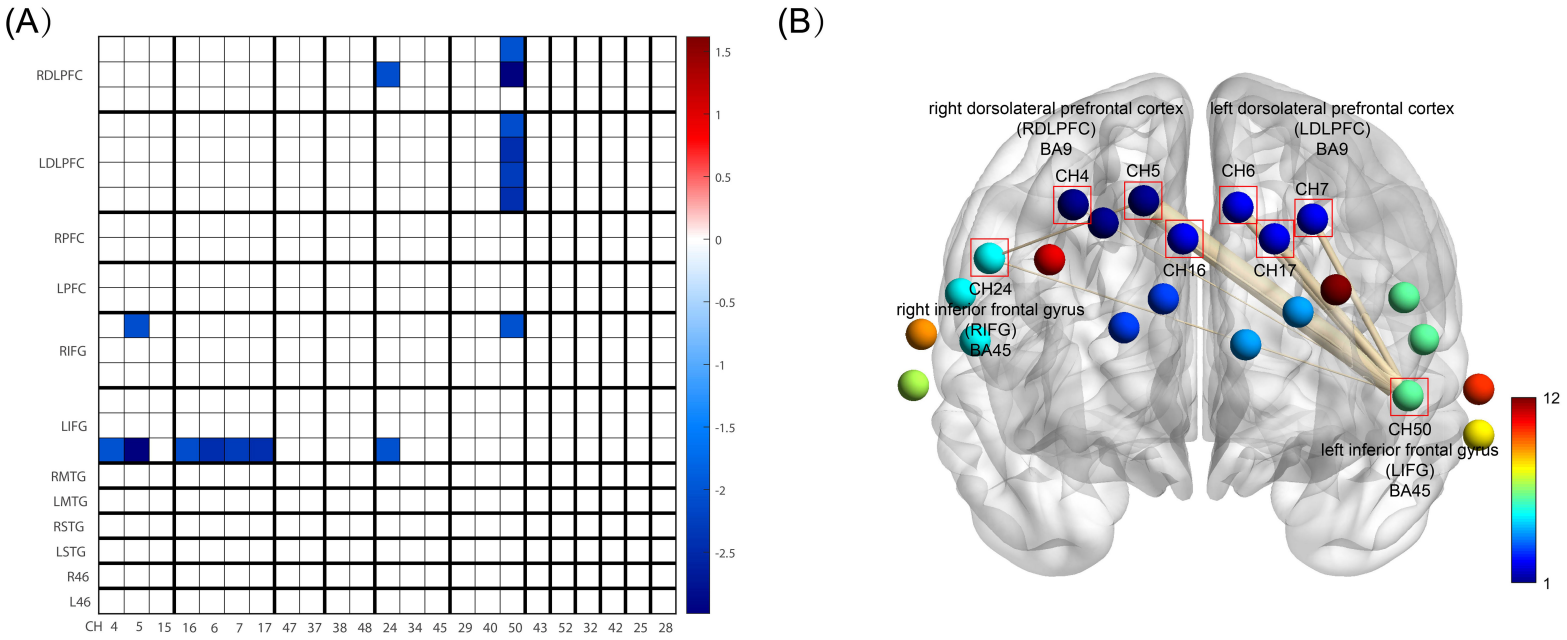
FC between HC and OCD group. **(A)** is FC Matrix. The horizontal axis shows the channel order, which is CH4, CH5, CH15, CH16, CH6, CH7, CH17, CH47, CH37, CH38, CH48, CH24, CH34, CH45, CH29, CH40, CH50, CH43, CH52, CH32, CH42, CH25, CH28 in sequence. The vertical axis represents the division of brain areas, which are, from top to bottom, the right and left dorsolateral prefrontal cortex (DLPFC), right and left frontal pole (FP), right and left inferior frontal gyrus (IFG), right and left middle temporal gyrus (MTG), right and left superior temporal gyrus (STG), and right and left BA46 (also DLPFC). These brain areas correspond to the channels on the horizontal axis, and each brain region includes its respective channels. The blue areas in the figure represent the significantly reduced functional connectivity in the HC group compared to the OCD group (p<0.05, not corrected for FDR), with darker the blue color, the smaller the p-value; **(B)** is BrainNet Viewer 3D Brain Connectomics, this figure visualizes the significantly different functional connections from Figure **(A)** in 3D. CH4, 5, and 16 all belong to the right dorsolateral prefrontal cortex, while CH6, 7, and 17 all belong to the left dorsolateral prefrontal cortex. The thickness of the lines connecting the points in the figure represents the magnitude of the difference, i.e., the p-value, with thicker lines indicating a greater degree of difference.

Analysis covered 12 brain areas through 23 channels, comparing functional connections (FC) between HC and OCD groups. Significant differences in FC were observed between CH5 (right dorsolateral prefrontal cortex, RDLPFC) and CH24 (right inferior frontal gyrus, RIFG) (*t*= -2.071, *p*= 0.043, not corrected for FDR), CH50 (left inferior frontal gyrus, LIFG) and CH4 (RDLPFC) (*t*= -2.021, *p*= 0.048, not corrected for FDR), CH50 (LIFG) and CH5 (RDLPFC) (*t*= -2.989, *p*= 0.004, not corrected for FDR), CH50 (LIFG) and CH16 (left dorsolateral prefrontal cortex, LDLPFC) (*t*= -2.085, *p*= 0.041, not corrected for FDR), CH50 (LIFG) and CH6 (LDLPFC) (*t*= -2.463, *p*= 0.017, not corrected for FDR), CH50 (LIFG) and CH7 (LDLPFC) (*t*= -2.291, *p*= 0.025, not corrected for FDR), CH50 (LIFG) and CH17 (LDLPFC) (*t*= -2.433, *p*= 0.018, not corrected for FDR), and CH50 (LIFG) and CH24 (RIFG) (*t*= -2.044, *p*= 0.045, not corrected for FDR) (**Table 2**). These results suggest that the functional connectivity between the aforementioned channels or brain areas in OCD may be greater than that in healthy controls (**Figure 5**).

**Table 2:** Difference of FC between HC and OCD group in RS.

Indicators	HC	OCD	*t*	*p*	effect size ^b^
FC between CH5 and CH24	0.434 ± 0.293^a^	0.558 ± 0.303	-2.071	0.043^*^	0.518
FC between CH50 and CH4	0.126 ± 0.307	0.273 ± 0.274	-2.021	0.048^*^	0.505
FC between CH50 and CH5	0.093 ± 0.278	0.290 ± 0.247	-2.989	0.004^*^	0.747
FC between CH50 and CH16	0.197 ± 0.303	0.349 ± 0.282	-2.085	0.041^*^	0.521
FC between CH50 and CH6	0.123 ± 0.285	0.285 ± 0.240	-2.463	0.017^*^	0.616
FC between CH50 and CH7	0.108 ± 0.312	0.273 ± 0.265	-2.291	0.025^*^	0.573
FC between CH50 and CH17	0.200 ± 0.292	0.366 ± 0.253	-2.433	0.018^*^	0.608
FC between CH50 and CH24	0.460 ± 0.302	0.626 ± 0.343	-2.044	0.045^*^	0.511

a Mean ± SD; ^b^For data that conform to a normal distribution, an independent-samples t-test is used, and Cohen’s d is calculated for the effect size; ^*^p< 0.05.

### 3.3 Comparison of IV, CV and number of word combinations between OCD and HC group in VFT

**Figure 6:**
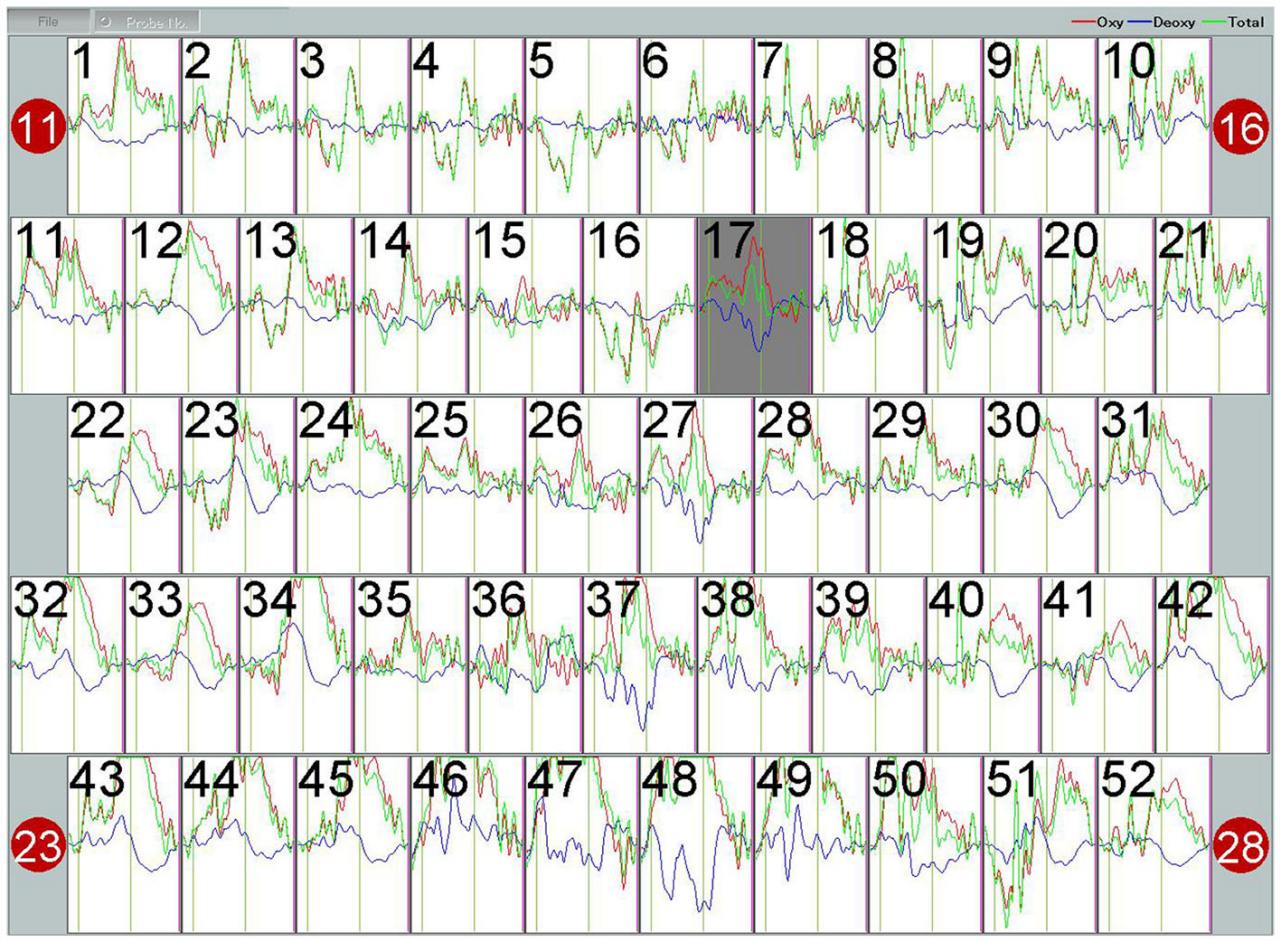
The hemodynamic response curve displayed on the fNIRS machine during the VFT performed by subject 04 in the OCD group represents the blood oxygen level. The red curve stands for Oxy, with the unit of (mmol/L)*mm. The waveform has undergone a 5-second moving average processing within the machine.

Analyzing the Integral Value (IV) and Centroid Value (CV) data produced by the ETG-4100 following the Verbal Fluency Task (VFT), significant differences were observed. Specifically, in the IV, notable disparities were evident in both the frontal lobe (*p*<0.001) and bilateral temporal lobes (*p*<0.001) between the healthy control (HC) and the OCD group, with the OCD group showing lower scores than the HC group. Regarding the CV, there were significant differences in the frontal lobe and bilateral temporal lobes between the HC group and the OCD group. As can be seen the CV values in the frontal lobe (*p*=0.026) and bilateral temporal lobes (*p*=0.029) of patients in the OCD group are significantly higher than those of the subjects in the HC group In the final comparison of VFT task performance, specifically in terms of the number of word combinations, the OCD group exhibited a significant decrease in the number of word combinations compared to healthy subjects (*p*<0.001) (**Table 3**). **Figure 6** shows the VFT result spectrum of one of the subjects in the OCD group. This spectrum was obtained from the ETG-4100 (fNIRS instrument).

**Table 3:** Difference of IV, CV and number of word combinations between HC and OCD group in VFT.

Indicators	HC	OCD	*t*	*z*	*p*	effect size ^c^
IV of the prefrontal lobe	130.45 (97.13,201.75)^a^	38.65 (3.68,66.83)		5.371	0.000^*^	0.671
CV of the prefrontal lobe	54.89 ± 7.94 ^b^	60.88 ± 12.55	-2.281		0.026^*^	0.570
IV of the Bilateral temporal lobe	193.54 ± 92.28	88.53 ± 85.57	4.720		0.000^*^	1.118
CV of the Bilateral temporal lobe	56.20 (52.35,59.28)	60.25 (55.48,64.65)		-2.182	0.029^*^	0.273
number of word combinations	13.81 ± 4.80	9.47 ± 3.58	4.101		0.000^*^	1.025

^a^Represented by the interquartile range (IQR) as [M (Q1, Q3)]. ^b^Mean ± SD. ^c^For data that conform to a normal distribution, an independent-samples t-test is used, and Cohen’s d is calculated for the effect size. For data that do not conform to a normal distribution, the Mann-Whitney U test is applied, and the effect size is calculated using r = z/√n; ^*^p< 0.05.

### 3.4 β comparison between OCD and HC groups in VFT

**Figure 7:**
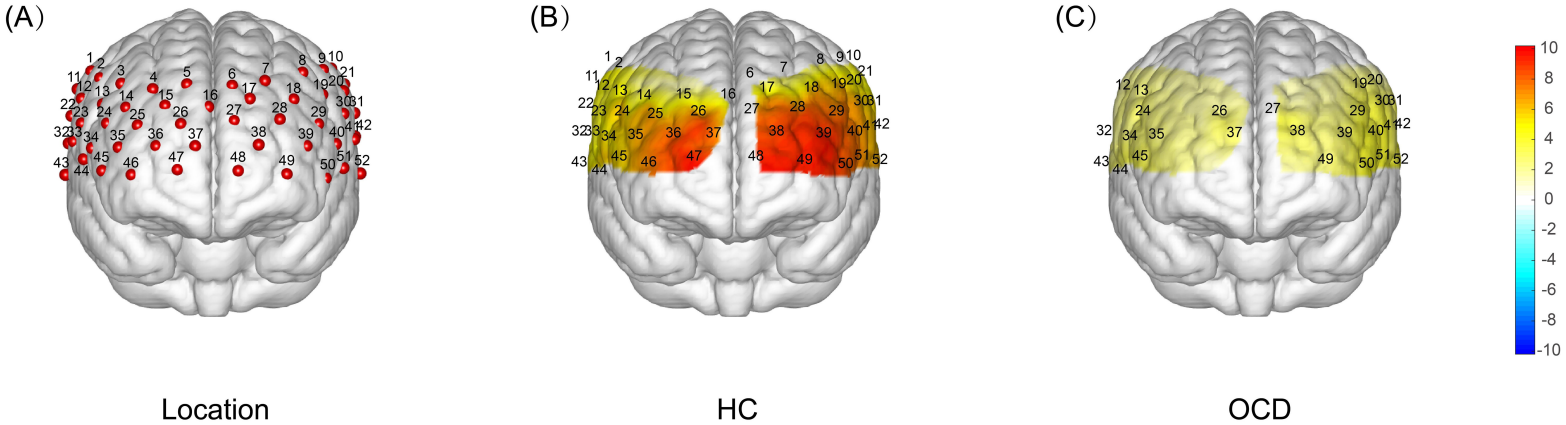
T-test Results of Brain Activation During VFT: HC vs OCD. **(A)** Localization map showing the 52-channel distribution across frontal and temporal lobes. **(B)** A one-sample t-test was conducted to compare the activation index β obtained during the VFT process in healthy subjects with 0. The resulting T-value brain map showed that the intensity of the red color represented the magnitude of the T-value, with a deeper color indicating a larger T-value and a higher degree of activation. The results indicated that there was a significant increase in activation in all channels except channels 3, 4, and 5. **(C)** A single-sample t-test was performed to compare the activation index β obtained from OCD patients during VFT with 0. This resulted in a T-value brain map, where the depth of the red color represents the magnitude of the T-value. The darker the color, the larger the T-value and the greater the degree of activation. The results showed that there was a significant increase in activation only in channels 12, 13, 19, 20, 24, 26, 27, 29, 30, 31, 32, 34, 35, 37, 38, 39, 40, 41, 42, 43, 44, 45, 49, 50, 51, and 52.

Analysis of each channel activation during the VFT revealed distinct neural activation between the HC and OCD groups. The HC group showed significantly higher β-values than 0 on all channels except channels 3, 4, and 5, indicating significant activation (all *p*<0.05). In contrast, the OCD group showed significant activation only in channels 12, 13, 19, 20, 24, 26, 27, 29, 30, 31, 32, 34, 35, 37, 38, 39, 40, 41, 42, 43, 44, 45, 49, 50, 51, and 52, where the β-values were significantly greater than 0 (all *p*<0.05). No significant difference from 0 was found in the remaining channels (*p*>0.05) (**Figure 7**).

Based on the locations, we mapped each area of interest to its corresponding functional brain area. Utilizing the relative concentration of oxy-Hb during the VFT in both OCD and HC group, we calculated the β for each area of interest(12 brain areas). Results presented in **Table 4** indicate a significant difference in neural activation between the OCD and HC group in Brodmann areas (BA) 9, 10, 21, 22, 45, and 46, across both frontal and temporal lobes, with a general trend of decreased activation levels in OCD group.

**Table 4:** Location of Brodmann area and β between HC and OCD group in VFT.

BA	Channel	HC-β	OCD-β	*t*	*z*	*p*	effect size ^c^
Right BA9	4, 5, 15, 16	0.0123(0.0086,0.0295)^a^	0.0027(-0.0027,0.0157)		2.309	0.021^*^	0.289
Left BA9	6, 7, 16, 17	0.0154 ± 0.0178 ^b^	0.0048 ± 0.0159	2.507		0.015^*^	0.627
Right BA10	37, 47	0.0341(0.0243,0.0515)	0.0111(-0.0094,0.0231)		4.592	0.000^*^	0.574
Left BA10	37, 38, 48	0.0340 ± 0.0181	0.0124 ± 0.0270	3.770		0.000^*^	0.943
Right BA45	24, 34, 45	0.0321 ± 0.0290	0.0116 ± 0.0196	3.311		0.002^*^	0.828
Left BA45	29, 40, 50	0.0367(0.0173,0.0534)	0.0120(0.0010,0.0300)		3.384	0.001^*^	0.423
Right BA21	43	0.0274(0.0111,0.0505)	0.0067(-0.0024,0.0195)		3.330	0.001^*^	0.416
Left BA21	52	0.0392 ± 0.0337	0.0159 ± 0.0212	3.305		0.002^*^	0.826
Right BA22	32	0.0249(0.0112,0.0496)	0.0081(-0.0044,0.0230)		2.873	0.004^*^	0.359
Left BA22	42	0.0298(0.0105,0.0422)	0.0084(-0.0003,0.0273)		2.820	0.005^*^	0.353
Right BA46	25	0.0318 ± 0.0253	0.0080 ± 0.0224	3.982		0.000^*^	0.996
Left BA46	28	0.0271(0.0174,0.0434)	0.0096(-0.0030,0.0194)		4.095	0.000^*^	0.512

^a^Represented by the interquartile range (IQR) as [M (Q1, Q3)]. ^b^Mean ± SD. ^c^For data that conform to a normal distribution, an independent-samples t-test is used, and Cohen’s d is calculated for the effect size. For data that do not conform to a normal distribution, the Mann-Whitney U test is applied, and the effect size is calculated using r = z/√n; ^*^p< 0.05. (BA9,46: Dorsolateral Prefrontal Cortex; BA10: Frontal Pole; BA45: Inferior Frontal Gyrus; BA21: Middle Temporal Gyrus; BA22: Superior Temporal Gyrus).

## 4 Discussion

In this study, we employed a 52-channel fNIRS device to monitor changes in relative hemoglobin concentrations during VFT and RS tasks among individuals with OCD and healthy controls. Our objective was to evaluate neural activation and FC across these tasks. During the VFT, we noted significant disparities between the OCD and healthy control groups in the IVs outputted by the device’s processing program, particularly within the frontal and bilateral temporal lobes. Specifically, IVs were reduced in the OCD group, while comparative analysis showed an elevation in CVs in the same cohort. Furthermore, a pronounced decrease in activation values was observed in frontal and temporal areas during task performance. Conversely, in the RS task, enhanced FC was identified between the left and right IFG and DLPFC in the OCD group relative to controls.

The VFT, as employed in fNIRS testing, emerges as a promising biomarker for distinguishing and diagnosing various emotional and mental disorders. IV and CV, indicative of cognitive activity state and brain reaction speed post-task initiation respectively, frequently diverge in mental health conditions such as depression, bipolar disorder, and schizophrenia when compared to healthy subjects (19). Analysis of IV and CV in OCD patients revealed a decrease in IVs, aligning with the diminished cognitive functions post-disease onset observed in these individuals (18). This diminished activation could result from the prefrontal and temporal lobes’ failure to adequately increase blood volume to compensate for the energy expended during the task. The notable rise in CVs, suggests a reduction or delay in reaction capability following task execution (19). Although VFT is considered a simple task, it still reflects the subject’s linguistic abilities, memory retrieval, and cognitive executive functions. During task performance, OCD patients may increase their decision-making time due to repetitive deliberations and cautious considerations, leading to delayed reactions. Additionally, their attention and cognitive resources may be diverted by obsessive ideas or impulses unrelated to the task, thereby reducing their focus and reaction speed on the task. Additionally, the detection of sawtooth-like waveforms in the OCD cohort (**Figure 6**), and the posterior shift in these waveforms due to increased CVs, presents a novel area for exploration (20). Further research is needed to determine if these changes can distinctly differentiate individuals with OCD from healthy controls.

This investigation further delineates the frontal and temporal lobes into distinct areas, namely the right and left DLPFC, the frontal pole, the IFG, the middle temporal gyrus, and the superior temporal gyrus, covering a total of 12 BA brain areas. The activation values in each of these areas were meticulously computed. Drawing from evidence in fMRI studies, the DLPFC and posterior areas have been implicated in the pathophysiology of OCD (50). Our findings indicate a reduction in activation values examined brain areas in comparison to HC. According to fNIRS principles, this reduction could stem from the challenges OCD patients face in achieving adequate blood supply to offset the oxygen consumption during the VFT, a compensatory mechanism essential for normal neuronal function. From the standpoint of brain functionality, Takeda and colleagues have demonstrated that OCD patients show impaired neurocognitive functioning and reduced frontal lobe activation during various cognitive tasks, including the VFT. Meanwhile, Takeda et al. also proposed a viewpoint that executing neurocognitive tasks necessitates the engagement of the prefrontal cortex (PFC), with the level of cognitive functioning influencing brain activation changes (51). Hence, the observed decrease in activation within the frontal and temporal lobes during the VFT in OCD patients is indicative of diminished neurocognitive capabilities. This is aligned with the observed decline in integral values and corroborates earlier studies on frontal lobe hemodynamic responses (26, 28).

Specifically, based on the selected brain areas of interest, we found that the activation of IFG, DLPFC in the frontal lobe and STG, MTG of the temporal lobe were significantly reduced compared with healthy subjects. Prior research has consistently reported functional decline of the IFG in OCD populations, including abnormalities in the white matter of the IFG and diminished activation in the left IFG among pediatric OCD patients (52, 53). OCD is associated with neurocognitive deficits, notably in executive function, verbal memory, verbal fluency, and processing speed, along with challenges in inhibiting cognitive and motor responses (22, 24). The IFG plays a pivotal role in the cognitive control network (CCN), crucial for inhibitory control during cognitive tasks and under stress (54). Thus, reduced IFG activation may reflect a functional deficit in cognitive control and inhibitory capabilities. Moreover, the DLPFC, integral to the executive system, has been consistently implicated in OCD’s pathophysiology (55). As part of the CSTC circuit (56), abnormalities in this region could trigger compulsive behaviors. In addition to the frontal lobe, In studies of obsessive-compulsive disorder (OCD) using fMRI and fNIRS, it has been proposed that there are abnormal activations in the temporal lobe areas, specifically in the STG and MTG, among OCD patients (20, 57). These two brain areas are a key component of the semantic system in the human brain. The insufficient activation of these two areas appears to be an additional finding resulting from the characteristic of the VFT task, which requires speaking. From the results of number of word combinations in the VFT, we found that the OCD group produced a lower number of word combinations compared to healthy subjects, suggesting a decline in their semantic system function. In addition, interestingly, some studies have pointed out that the thought suppression is mediated by MTG (58). This may suggest that activation disorders in these temporal lobe areas are related to thought suppression disorders in obsessive-compulsive disorder (OCD), but further research is needed.

In the realm of RSFC, our study examined connectivity across all channels within the 12 brain areas during the RS task. The analysis revealed significant increases in FC between the left IFG and bilateral DLPFC, left IFG and right IFG, and right IFG and right DLPFC (without FDR correction). Although we are able to detect differences in functional connectivity between the two groups of subjects, we cannot overlook the fact that the differences did not pass the FDR correction. The reasons for this could be attributed to an insufficient sample size or the large number of channels(number of 23) we focused on. Therefore, our functional connectivity results indicate the possibility of differences between the groups, albeit with caveats.

Research on OCD patients has shown increased intrinsic connectivity between the bilateral insula and bilateral precuneus gyrus, extending to the inferior parietal lobule and SMA (59). This suggests heightened connectivity within executive and attention networks in OCD, with the severity of the disease positively correlating to this increase in FC. Meanwhile, OCD patients have been shown to display increased spontaneous FC in the bilateral IFG, extending to the bilateral insula and the bilateral medial prefrontal cortex/anterior cingulate cortex (mPFC/ACC) (60). Enhanced FC between the IFG and the anterior supplementary motor area (SMA) in OCD patients suggests a possible mechanism for the observed abnormal activation during response inhibition tasks (61, 62). Although the SMA was not included in the areas of interest for this study, we noted a rise in FC between the IFG and DLPFC, aligning with prior observations of DLPFC dysconnectivity in resting-state FC analyses (63). Thus, the IFG and DLPFC abnormalities, along with their increased connectivity, may play a crucial role in OCD’s pathogenesis. This study’s most significant FC enhancement was between the left IFG and DLPFC. While the right IFG of OCD patients has been reported as the primary area implicated in response inhibition and affected VFT performance by participating in remote associate thinking (64, 65), the left IFG’s involvement in motor response inhibition is also supported (66). Our findings underscore the importance of both IFG and DLPFC in response inhibition mechanisms. Furthermore, in the context of transcranial magnetic stimulation (TMS) treatment for OCD, targeting the right DLPFC has shown clinical benefits (67–69).

On the other hand, our FC results contrasts somewhat with prior resting-state fMRI studies, which have documented specific connectivity disruptions in OCD, such as excessive striatum-cortical network connectivity, diminished thalamus-striatum (caudate and putamen) connectivity, and reduced anterior cingulate cortex (ACC) connectivity with frontal-parietal networks (30). Additionally, variations in CSTC circuit connectivity have been correlated with OCD symptom severity (70). Geffenn et al. proposed that diminished connectivity between the salience network (SN) and the default mode network (DMN) might serve as an OCD biomarker (71). The observations from this study contribute to a growing body of research on FC in OCD

There are still some limitations in this study. Firstly, the relatively small sample size resulted in our RS functional connectivity results not passing the FDR correction. Secondly, this study did not fully distinguish between patients with first-episode OCD and those under medication. Although we tried to include patients who were diagnosed with OCD for the first time during the actual recruitment of OCD patients, among the 32 OCD patients, two were already taking SSRI medications during the fNIRS testing due to the late scheduling of the test. We have uploaded the comparative results between the OCD group (excluding these two patients) and the HC group in the **Supplementary Materials**. While the results are almost identical to those mentioned above, we still need to report them. Additionally, the study’s focus was narrow, excluding other psychiatric disorders for comparative analysis and not incorporating measures of OCD symptom severity. This omission restricts the potential to explore the correlation between brain activation, functional connectivity changes, and the nuances of OCD symptoms. Also, the lack of subgroup analysis on the spectrum of obsessive thoughts and compulsive behaviors leaves room for further investigation. Despite these limitations, the study underscores significant neurocognitive impairment in OCD patients, as evidenced by decreased functional activation in frontal and temporal areas during the VFT. The increased FC between the IFG and DLPFC, especially from the left IFG to the right DLPFC, may underline the neural mechanisms behind executive dysfunction and response inhibition deficits in OCD, suggesting potential pathways for future therapeutic interventions and research.

## Data Availability

The raw data supporting the conclusions of this article will be made available by the authors, without undue reservation.
