# Supplementary material for: Neurological activation during verbal fluency task and resting-state functional connectivity abnormalities in obsessive-compulsive disorder: a functional near-infrared spectroscopy study
Source: Front Psychiatry. 2024 Aug 30;15:1416810. doi: 10.3389/fpsyt.2024.1416810 (PMC11392768; doi:10.3389/fpsyt.2024.1416810)
Supplement: Supplementary file 1 [file DataSheet1.pdf]

This supplementary materials shows the statistical results related to 32 people in the healthy subjects group and 30 people in the OCD group (excluding 2 people with medication history):

Table 1. Comparison of clinical characteristics between HC group and OCD group

Indicators	HC(n=32)	OCD(n=30)	*χ2*	*z*	*t*	*p*
Male	14	17	1.033			0.309
Female	18	13				
Age(years)	22.50(21.00,26.50) [a]	22.00(18.00,29.00)		0.600		0.548
Education(years)	15.00(14.25,16.00)	14.50(11.00,16.00)		1.741		0.082
Y-BOCS scores	3.25±1.44 [b]	17.93±5.94			-13.569	0.001 *
SSS score	2.00(1.25,3.00)	3.00(2.00,3.00)		-0.733		0.464

[a] Represented by the interquartile range (IQR) as [M (Q1, Q3)];

[b] Mean ± SD;

* $p< 0.05$.

Table 2. Difference of FC between HC and OCD group in RS

Indicators	HC	OCD	*t*	*p*	effect size [b]
FC between CH5 and CH24	0.434±0.293 [a]	0.612±0.284	-2.424	0.018 *	0.616
FC between CH50 and CH4	0.126±0.307	0.282±0.281	-2.080	0.042 *	0.529
FC between CH50 and CH5	0.093±0.278	0.308±0.242	-3.227	0.002 *	0.820
FC between CH50 and CH16	0.197±0.303	0.368±0.280	-2.299	0.025 *	0.584
FC between CH50 and CH6	0.123±0.285	0.299±0.237	-2.629	0.011 *	0.668
FC between CH50 and CH7	0.108±0.312	0.290±0.259	-2.490	0.016 *	0.633

FC between CH50 and CH17	0.200±0.292	0.389±0.238	-2.781	0.007*	0.707
FC between CH50 and CH24	0.460±0.302	0.598±0.298	-1.797	0.077	0.457

[a] Mean ± SD; [b]For data that conform to a normal distribution, an independent-samples t-test is used, and Cohen's d is calculated for the effect size. $^{*}p< 0.05$.

Table 3. Difference of IV, CV and number of word combinations between HC and OCD group in VFT

Indicators	HC	OCD	*t*	*z*	*p*	effect size[c]
IV of the prefrontal lobe	130.45 (97.13,201.75)[a]	38.05 (-0.05,64.68)		5.494	0.000*	0.698
CV of the prefrontal lobe	54.89±7.94[b]	61.09±12.95	-2.287		0.026*	0.581
IV of the Bilateral temporal lobe	193.54±92.28	84.89±87.18	4.758		0.000*	1.209
CV of the Bilateral temporal lobe	56.20 (52.35,59.28)	60.25 (55.63,64.98)		-2.247	0.025*	0.285
number of word combinations	13.81±4.80	9.27±3.49	4.238		0.000*	1.077

[a] Represented by the interquartile range (IQR) as [M (Q1, Q3)]; [b]Mean ± SD; [c]For data that conform to a normal distribution, an independent-samples t-test is used, and Cohen's d is calculated for the effect size. For data that do not conform to a normal distribution, the Mann-Whitney U test is applied, and the effect size is calculated using $r = z/\sqrt{n}$; $^{*}p< 0.05$.

Table 4. location of Brodmann area and β between OCD and HC group in VFT.

BA	Channel	HC-β	OCD-β	*t*	*z*	*p*	effect size[c]
Right BA9	4、5、15、16	0.0123(0.0086,0.0295)[a]	0.0030(-0.0028,0.0174)		2.113	0.035*	0.268
Left BA9	6、7、16、17	0.0154±0.0178[b]	0.0053±0.0163	2.319		0.024*	0.589

Right BA10	37、47	0.0341(0.0243,0.0515)	0.0132(-0.0064,0.0244)		4.367	0.000*	0.555
Left BA10	37、38、48	0.0340±0.0181	0.0145±0.0266	3.409		0.001*	0.866
Right BA45	24、34、45	0.0321±0.0290	0.0115±0.0200	3.237		0.002*	0.823
Left BA45	29、40、50	0.0367(0.0173,0.0534)	0.0127(-0.0003,0.0318)		3.212	0.001*	0.408
Right BA21	43	0.0274(0.0111,0.0505)	0.0067(-0.0027,0.0163)		3.367	0.001*	0.428
Left BA21	52	0.0392±0.0337	0.062±0.0219	3.168		0.002*	0.895
Right BA22	32	0.0249(0.0112,0.0496)	0.0081(-0.0064,0.0203)		2.888	0.004*	0.367
Left BA22	42	0.0298(0.0105,0.0422)	0.0084(0.0020,0.0284)		2.564	0.010*	0.326
Right BA46	25	0.0318±0.0253	0.0092±0.0226	3.700		0.000*	0.940
Left BA46	28	0.0271(0.0174,0.0434)	0.0010(-0.0019,0.0215)		3.845	0.000*	0.488

[a] Represented by the interquartile range (IQR) as [M (Q1, Q3)]; [b]Mean ± SD; [c]For data that conform to a normal distribution, an independent-samples t-test is used, and Cohen's d is calculated for the effect size. For data that do not conform to a normal distribution, the Mann-Whitney U test is applied, and the effect size is calculated using $r = z/\sqrt{n}$; $^{*}p < 0.05$.

(BA9,46: Dorsolateral Prefrontal Cortex; BA10: Frontal Pole; BA45: Inferior Frontal Gyrus; BA21: Middle Temporal Gyrus; BA22: Superior Temporal Gyrus.)
